# A qualitative study of prescribing errors among multi-professional prescribers within an e-prescribing system

**DOI:** 10.1007/s11096-020-01192-0

**Published:** 2020-11-09

**Authors:** Fahad Alshahrani, John F. Marriott, Anthony R. Cox

**Affiliations:** 1 School of Pharmacy, Institute of Clinical Sciences, College of Medical and Dental Sciences, University of Birmingham, Birmingham, UK; 2 Security Forces Hospital, Riyadh, Saudi Arabia

**Keywords:** Clinical pharmacy, CPOE, Electronic prescribing, Prescribing

## Abstract

*Background* Computerised Physician Order Entry (CPOE) is considered to enhance the safety of prescribing. However, it can have unintended consequences and new forms of prescribing error have been reported. *Objective* The aim of this study was to explore the causes and contributing factors associated with prescribing errors reported by multidisciplinary prescribers working within a CPOE system. *Main Outcome Measure* Multidisciplinary prescribers experience of prescribing errors in an CPOE system. *Method* This qualitative study was conducted in a hospital with a well-established CPOE system. Semi-structured qualitative interviews were conducted with prescribers from the professions of pharmacy, nursing, and medicine. Interviews analysed using a mixed inductive and deductive approach to develop a framework for the causes of error. *Results* Twenty-three prescribers were interviewed. Six main themes influencing prescribing were found: the system, the prescriber, the patient, the team, the task of prescribing and the work environment. Prominent issues related to CPOE included, incorrect drug name picking, default auto-population of dosages, alert fatigue and remote prescribing. These interacted within a complex prescribing environment. No substantial differences in the experience of CPOE were found between the professions. *Conclusion* Medical and non-medical prescribers have similar experiences of prescribing errors when using CPOE, aligned with existing published literature about medical prescribing. Causes of electronic prescribing errors are multifactorial in nature and prescribers describe how factors interact to create the conditions errors. While interventions should focus on direct CPOE issues, such as training and design, socio-technical, and environmental aspects of practice remain important.

**Electronic supplementary material:**

The online version of this article (10.1007/s11096-020-01192-0) contains supplementary material, which is available to authorized users.

## Impacts on practice

- When implementing e-prescribing a system-wide review of prescribing, including characteristics and behaviours of prescribers, patients and the work environment should be undertaken to design out error.
- Prescribers from all professions describe similar difficulties with electronic prescribing, so training provision should be provided for all and should be multi-professional.
- Those implementing electronic prescribing systems should warn users of the distinct new forms of prescribing error that can occur.

## Introduction

Medication errors, defined as ‘a failure in the treatment process that leads to, or has the potential to lead to, harm to the patient’ [1], are responsible for significant morbidity and mortality, as well as increased costs of healthcare [2, 3]. The World Health Organisations Third Global Patient Safety Challenge is focused on a reduction in medication errors by 50% [4] and reducing prescribing errors is a key part of this drive. In the UK, prescribing errors are estimated to occur in 8.9 to 14.7% of hospital inpatients and discharge medications [5–7].

Computer Physician Order Entry systems (CPOEs) are seen as essential for improving both efficiency and patient safety in relation to prescribing [2, 8–10]. CPOE enables prescribers to enter drug prescriptions via a computer application rather than paper. Evidence suggests that the use of CPOE reduces the prevalence of prescribing errors, [11–15] by removal of legibility issues, guiding prescribers to appropriate prescribing decisions, and providing a robust system of audit.

Although the overall error reduction from CPOEs is uncontroversial, novel types of error have emerged associated with CPOE [16–18]. Some case studies of CPOE implementation have showed an increase in the number of higher severity medication errors [19, 20]. Malfunctions of clinical decision support systems (CDSS) in CPOE can also impact on patient safety [21] and can arise from the complexity of coding clinical concepts in hierarchies within CPOE [22]. Staff perceptions of prescribing safety following CPOE introduction may also be counter-intuitive. Davies et al. [23] found that health care staff’s perception of safety culture deteriorated after electronic prescribing was introduced. Despite this, CPOE remains a key part medication error prevention [24].

CPOE design choices can also make some errors more likely; e.g. as a result of an incorrect selection on a drop-down menu, an electronic prescription for diamorphine was created at 70 times the required dosage [25]. Similar unintended adverse consequences of CPOE systems have been reported [16, 26, 27].

Qualitative research on prescribing errors has examined multiple influences of prescribing errors in secondary care [5, 28–30], highlighting insufficient training and high workloads. A meta-synthesis of integrating CDSS into clinical work found problems with useability and socio-technical issues on implementation [31].

In many countries non-medical prescribing is becoming more common [32], but there are no qualitative studies of prescribing errors by medical and non-medical prescribers in the context of CPOE.

### Aim of the study

The study aimed to examine the views of pharmacists, nurses, and medical prescribers on the causes of electronic prescribing errors in a CPOE system in one large, multi-speciality UK NHS hospital.

### Ethical approval

The study was approved by the University of Birmingham Research Ethics Committee (ERN_15-0161), Research and Development Department at the University Hospitals Birmingham NHS Foundation Trust (UHBFT) in September 2016 and NHS ethics committee.

## Method

### Design

The study used a qualitative design, employing semi-structured interviews exploring the potential causes and contributing factors of prescribing errors in an electronic prescribing context. Thematic content analysis was combined with critical incident technique (CIT), to explore the factors influencing prescribing errors. CIT allows interviewees to describe an event, allowing collection of facts, and subsequent evaluation of a cause. It is well suited to studying the interaction of subjects with technology, having been first developed with pilots [33]. CIT informed the interview guide, as well as prior studies investigating prescribing errors [6, 28]. Three case studies of prescribing errors were used to provoke discussion of error in a non-threatening manner, since participants might not be willing to talk comfortably about errors of their own or others owing to fear of incrimination. The final interview guide (See Supplement 1) was piloted with four experienced pharmacists to ensure clarity and face validity. The interview guide also examined prescribers’ specific views on CPOE design; these results will be reported elsewhere. The analysis of the views and experience of three groups of prescribers, with differing standpoints, additionally provided a form of data triangulation.

### Study location

Participants were recruited from an academic tertiary care hospital in the West Midlands, which operates a locally developed electronic prescribing system (Patient Information Communication System—PICS). The system includes integrated clinical decision support features, such as dose range checking, drug interactions alerts and contraindications (e.g. drug-disease, allergies). The system is used for in-patient, out-patients, and day care prescribing.

### Participants

A purposive sampling methodology was used in order to target varied groups of prescribers. All active medical and non-medical licensed prescribers (pharmacists and nurses) working in the trust were eligible for inclusion.

The prescriber types approached in this study were:

- Junior doctors: All training and non-training grades (Foundation Year 1 and Foundation Year 2, Specialty Registrars, Junior Specialist Doctors).
- Senior doctors: Staff Grades and Consultants.
- Independent Pharmacist Prescribers.
- Independent Nurse Prescribers.

All medical and non-medical prescribers in the hospital were contacted via email and invited to interview. All prescribers who expressed an interest received an email consisting of an invitation letter and participant information leaflet. It was made clear to participants that any individuals involved in errors should not be named, and that no blame would be assigned to them as a result of the interviews. Interviews were arranged at a mutually convenient time and place for the interview, in a workplace setting (such as a quiet office). No third parties were present at interviews. Consent was obtained before the interview commenced and participants were provided with a brief explanation of the purpose of the study.

### Data collection

The face to face interviews were conducted between 5th December 2016 and 25th April 2017. Interviews were recorded and discussions lasted between 20 and 30 min. Interviews were conducted by a male investigator (FA), a qualified pharmacist of several years clinical experience undertaking a fulltime PhD in electronic prescribing. The interviewer had no prior relationship with any of the participants. Participants were not given transcripts for correction.

### Data analysis

Interview data was transcribed verbatim into an anonymous format, which was loaded into NVivo® version 10 for data management. Data analysis and recruitment was conducted in parallel, with ongoing analysis informing the researcher. A response rate could not be calculated there was no reliable figure for the total number of prescribers within the organisation. However, participants were recruited until data saturation was achieved, when it was judged that no new additional themes were arising from the analysis [34].

An inductive and deductive approach was used in this study, to develop a thematic analysis of the data. Inductively, this was based on the interviewees' responses. The deductive approach was based on a framework for analysing risk and safety in clinical medicine [35] which is based on frameworks used in the human factors field. It includes institutional contexts, organisational factors, the work environment, team and individual factors, task factors, and patient characteristics. Coding was carried out on a line by line analysis. All transcripts were coded by FA. Following initial analysis, codes were refined and combined where appropriate, and clustered into broad themes. Coding accuracy and thematic analysis was cross-checked by two additional researchers (ARC, JFM), with differences resolved via consensus.

## Results

### Characteristics of the participants

A total of 23 medical and non-medical prescribers were interviewed. No one who responded to the initial study email refused to participate. We were unable to estimate a response rate, since we have no reliable figure for the total number of prescribers within the organisation. The demographics of the study participants are in Table 1.

**Table 1:** Summary of study participants

Group	Number	Gender
Senior doctors	5	5 male
Junior doctors	5	2 male, 3 female
Nurse prescribers	6	6 female
Pharmacist prescribers	7	3 male, 4 female

### Participants’ perspectives on prescribing errors

**Fig. 1:**
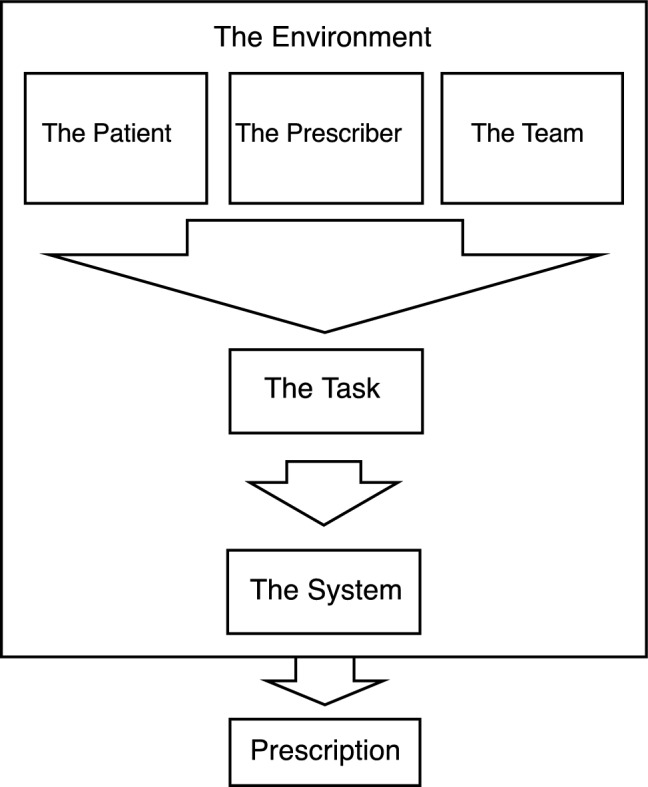
Conceptual figure of the interaction of the six major themes leading to the production of a prescription

Six major themes influencing prescribing errors in CPOE systems emerged from the analysis, which are illustrated in the conceptual framework in Fig. 1. While these emergent themes were clear, they were interconnected, with participants describing a complex process during the process of prescribing. Multiple causes could contribute to a prescribing error. The following sections describe these major themes.

### The system

Issues related to the CPOE design were common. Selection of the wrong item from an electronic dropdown list was identified by most participants (18/23). These included juxtaposition errors, where a medication with an entirely different indication than intended, listed before or after the desired medication, is erroneously chosen. Prescribers felt that such issues would not happen when using handwritten order systems.

*it’s easy to click on the wrong drug, not double check it and just click again and obviously you are responsible for that’s being tracked, uh, and I think when you are doing written prescribing, you know what you’re writing. Whether you write it fast or slow, you know what you’re writing…”*

**Junior Doctor 2**

Prescribers also noted that on entering the first few letters of a drug name, the system would “suggest” a medicine name, which could be easily selected in error. Prescribers suggested the imprecision of touch screen computers worsened this risk.

Prescribers noted that errors can occur by selecting the wrong patient owing to the nature of the patient list structure; the correct medication being prescribed to the wrong patient (which was also linked to prescribing away from the physical location of the patient). The ability to prescribe for patients remotely rather than attending the patient directly, was also believed to lead to a disconnect between the act of prescribing and clinical assessment of the patient.

*if you prescribe something without assessing the patient, so if someone rings and says oh, they need some painkillers, you might prescribe a painkiller that they shouldn’t have, based on their clinical picture.*

**Junior Doctor 3**

Auto-population of information by the CPOE, such as drug dosing frequencies, to a default setting, led to prescribers allowing defaults stand when inappropriate to the specific patient. Not correcting them led to reduced conflict with the CPOE system. An example of prescribing error from auto-population was:

*I had a patient that was prescribed baclofen. We changed it from tablet to oral solution. They were on 10 mg once a day before they changed it, so it was prescribed fine on PICS, baclofen 10 mg tablets once a day. But they had swallowing difficulty, so we changed it to oral solution which defaulted the dose to 5 mg of TDS.*

**Senior Doctor 2**

Participants described an expectancy that CPOE’s safety measures would intervene to prevent errors, that they felt made them complacent. Yet, at the same time prescribers described over-riding alerts without conscious jugement. This was due to a perception of too many warnings and alert fatigue.

*After a while, you get used to the warnings, so you, sometimes, you probably don’t read them as well as you should so you just keep clicking the warning off and you might miss a warning and still prescribe a drug for a patient that probably shouldn’t be having it.*

**Junior Doctor 3**

Prescribers noted the complex nature of electronic prescribing systems and how the rigidity of the process of prescribing medicines has the potential to lead to errors. This was noted particularly for non-standard intravenous products.

### The prescriber

Participants noted a lack of knowledge including deficits in drug knowledge for appropriate drug dosing (e.g., giving the wrong dose for renal or older patients) and failure to apply a protocol (e.g. modifying the dose in the presence of renal failure).

*there would have needed to be a knowledge by the prescriber about the correct dosing of the enoxaparin and really you should not need a computer to…I would expect a doctor in renal medicine to know what the correct dose was and prescribe the correct dose.*

**Nurse 2**

Participants reported that high work load caused rushed working practices that led to prescribing errors. Prescribers’ emotional status and their stress levels and tiredness were described by them as being likely to contribute to an error.

*if the doctor is very busy and particularly they’re on call and they have got a lot of things to do; they tend to forget things, we’re human beings and we tend to forget things and when you forget, you make errors.*

**Senior Doctor 1**

Participants reported that errors were caused by slips in attention or lapses of memory. Memory lapses included situations such as prescribing a medication to which a patient has an allergy. Forgetting to navigate from the current patient’s profile to prescribe for another patient was reported as an example of slips.

* I saw four patients. The nurse comes to me. I’ve got PICS opened up and nurse tells me that your patient has got a heart rate of 200. So I want to prescribe a beta-blocker. Then I look for the patient but I usually don’t concentrate because I’m still stuck with another patient. In the, doing the work, I’m looking after another patient and I’m prescribing medicine in another. So I ended up prescribing a medicine for my patient, the one who I was looking after now. So these are very common errors I see.*

**Senior Doctor 2**

Negligence in following standard procedures was identified as a contributing factor to prescribing errors. Those prescribers accustomed to a paper based system were incautious within an e-prescribing system.

*it's because people don't understand how electronic prescribing system works, they don't know how to read and follow through because we are so used to paperwork and people are not really, I hate to say this, but people do things without even reading exactly what they are doing, that could cause an error.*

**Nurse 1**

### The patient

Patients with complex comorbidities receiving multiple medications were cited by many participants as a high risk area for prescribing. These could include issues involving routes of administration, as well as dose and drug choice.

*on the critical care particularly the routes that are normally available on the ward are not always appropriate for my patients. So, something that they were previously swallowing is now going down a nasogastric tube – so, we have to make a lot of dose adjustments for going between IV and oral routes, or oral and other enteral routes, so there are quite a lot of errors there”*

**Pharmacist 5**

Errors arising from mis-remembered medication history from patients was also raised.

*Sometimes patients think they’re on a specific dose, but they’re not on that dose, so you might go from patient information and then you prescribe the wrong dose, but it’s on what the patient said as well.*

**Junior Doctor 3**

Prescribing errors were reported to occur when a medication history is unavailable or irretrievable when patients are admitted to hospital.

*it becomes a detective case and, and again, that’s not a very safe way of prescribing because, but, you know, the …You're faced with, out of hours you're faced with the option of not prescribing any drugs—and waiting for the next day until the GP surgery opens or a family member can bring in the prescription.*

**Junior Doctor 4**

Prescribers reported how unfamiliarity with the patients contributed to their prescribing errors, even in the presence of a good medical history. Treating a patient under the care of others while oncall was a common example given:

*You’re asked to prescribe this or, you are asked to see them because they’re deteriorating, you want to prescribe this or that, and you don’t know them as well. ***Junior Doctor 2.**

### The task

The task of prescribing was a strong theme. Prescribers noted that errors occur because a medication history is unobtainable, especially after business hours. During these periods GP surgeries are closed and the patient could be unable to provide an accurate medication history. Lack of access to patient records were identified as a contributing factor leading to prescribing errors.

*the doctors don’t have access to summary care records so it’s hard for them to get a drug history properly especially if a patient comes in when they’re confused….I’ve seen that sometimes patients say oh, I’m on bisoprolol but they don’t know the dose, so the doctors just prescribe bisoprolol and they just go with the default on PICS”*

**Junior Doctor 4**

Prescribers noted that some medications (such as morphine or HIV medications) are not shown on patient records as they may be prescribed by specialists, which could lead to medication omissions. The difficulties of sharing or transferring information between hospital and primary health care sectors was noted. Outdated discharge summaries and modifications to therapy by GPs or out-patient prescribers that had not been updated in care records were cited as specific examples of causes of inaccurate medication histories, inevitably associated with prescribing error.

*I mean there is always the barrier in certainly between community and hospital. We don’t have access to their records, they don’t have access to our records.*

**Junior Doctor 4**

Prescribers also noted that errors can occur when they want to prescribe a medication in the absence of essential laboratory results.

*So sometimes, um, you’ll prescribe a medication before the blood results are back.[.] You might put them on a diuretic before they know they’ve got acute kidney injury.*

**Senior Doctor 4**

### The team

Several prescribers highlighted poor communication between team members. As an example, using bed numbers rather than patient’s name was reported to cause prescribing errors. Inadequate communication between healthcare professionals when patients were being transferred, or during care team hand-over, was noted as a cause of prescribing errors.

*if someone has told you—so you mix them up, yeah, so if someone has told you bed 4 needs paracetamol and bed 5 needs codeine, you might mess it up. Especially when they don’t use names, if they use bed numbers, you could mix it up because it is a pressured environment”*

**Junior Doctor 3**

Staffing levels were also mentioned as being associated with prescribing error. Inadequate staff numbers, staff turnover and providing cover for absent colleagues were highlighted to increase workload and thus predispose to error generation.

### The environment

Interviewees suggested that the working environment is a major contributor to prescribing error. Heavy workload, time pressures, a chaotic, distracting environment and the need to perform more than one task simultaneously, in the context of the CPOE system, were commonly mentioned.

### On differences between non-medical and medical prescribers

This study involved participants from across three differing prescribing professions and found no systematic differences in the experiences of CPOE based on professional background. Although pharmacists’ role as a clinical reviewer of other professions’ prescribing led them to volunteer examples of other professions’ prescribing practice, reflections on their own prescribing practice was similar to that of other professions.

## Discussion

Our study found that the causes and contributing factors to electronic prescribing errors described by prescribers from different professions are multifactorial and interconnected. They have been classified into six high-level categories (the computer system, the prescriber, the patient, the task, the team and the work environment) that contributed to prescribing errors. The causes and contributing factors of electronic prescribing errors reported from different prescribers (medical and non-medical) were similar to many of the prescribing errors that occur with conventional handwritten prescribing [5, 28, 29] with the addition of errors related to the electronic system specifically. A qualitative study of implementing CDSS, rather than CPOE, found some similar categories, including issues such as people, culture, communication, as well as the more technical issues one might expect [36]. Implemention of CPOE needs at least as much thought put into the human and organisational implementation, as it does the technical implementation.

### Types of errors

Electronic prescribing systems reduce prescribing errors overall and they can create or propagate new issues which have been highlighted in previous studies [18, 37–39]. Our study confirms this with medical and non-medical prescribers. Whether these errors arise from design interface problems resulting from densely populated medication lists causing juxtaposition problems, prescribers relying on potentially inappropriate default doses, or human factor considerations, the recognition of such problems and their consequences by system designers should improve outcomes when implementing new alerts in e-prescribing systems. Inflexible or complex ordering processes made prescribing particularly difficult and users noted that this could result in forced errors.

### Distractions and over-reliance

Also, distracting features of electronic systems caused by excessive alerts generated by the system during prescribing were cited as a disruptive effect by prescribers. A series of best practices have been suggested for alerts in CPOE and CDSS systems [40], but the evidence base for optimal amount of alerts and nature of such alerts, is weak [41].

Prescribers in our study also noted the potential for over-reliance on CPOE systems. Such automation bias has been studied in experimental conditions and over-reliance on CDSS in CPOE led to increased prescribing errors [42].

### Prescriber knowledge and training

A lack of knowledge of medication appears to be a major contributor to prescribing errors in previous studies [5] and arises in our study. Addressing knowledge deficits has been a long term concern [43], with continuing professional education for safe prescribing practice essential. Online resources such as the eLearning programme tool called Standard Computerised Revalidation Instrument for Prescribing and Therapeutics (SCRIPT) [44] can promote safer prescribing in both medical and non-medical prescribers (https://www.safeprescriber.org).

Hospitals should ensure all users have access to adequate training before accessing the system. Learning outcomes for the electronic prescribing records have recently been published [45], and similar is required for electronic prescribing systems. A literature review of the training offered in electronic prescribing to qualified prescribers found only seven papers, which rarely covered the potential downsides of electronic prescribing [46].

However, training may not always be effective. A small randomised control trial of feedback and training in relation to electronic prescribing found little effect and the authors argued that re-designing electronic prescribing systems would change prescriber behaviour more than education [47]. Latent failures built into CPOE provide the conditions in which prescribing errors occur, when high work load pressure and working environment distractions appear [48]. However, compulsory condensed technical training on how to use the system effectively, particularly during the “shakedown” phase of implementation would seem prudent [49].

### Socio-technical issues

Our study found prescribing errors related to the hierarchy, culture and poor communication between team members. These errors were mainly owing to barriers in communication between healthcare professionals and the inability to access insufficient drug information and guidelines at the time of prescribing. Prior research on the effect of CPOE on pharmacist-physician communication has shown increased frequency of communications between pharmacists and physicians [50] provoked by many of the causes of error (such as lack of knowledge of the prescribing system) was also found in our study. A systematic review of the implementation of CDSS found similar socio-technical issues to those we found, including communication issues [51].

### A complex problem

Our study indicated that inadequate access to a medication history of patients across health care sectors leads to prescribing errors. Franklin et al. [6] found that a lack of information of patients’ medication histories from primary care settings contributed to prescribing errors in hospital settings. Significant improvements in medication histories and documentation of allergies has been shown when pharmacists are given this responsibility [52].

Our finding that medical and non-medical prescibers described prescribing errors as multifactorial is consistent with previous studies [5, 29, 30, 53, 54]. All of these factors would be common to different prescribing professions and all would be subject to the same human cognitive biases, so it is perhaps not surprising that little difference between professions in the experience of CPOE was apparent.

### Reducing prescribing errors

Recommendations for implementation of CPOE have been published [55, 56] and it is clear that socio-technical changes to interprofessional and patient communications caused by CPOE are also an outcome of CPOE implementation. Many issues are common to both CPOE and paper-based prescribing systems. We did not distinguish between these, since this is not a comparative analysis. We argue that CPOE systems should be examined as a whole, and it can be difficult to make the judgement about whether the CPOE was or was not involved in any particular event.

A policy brief summary analysing 40 systematic reviews, suggested actions dealing with prescribing error, including education for prescribers, incorporating computerised alerts, incorporating tools to guide prescribing, and encouraging multidisciplinary teams, including pharmacists, to care for patients [57]. This underlines that CPOE is only one intervention to reduce prescribing errors.

### Strengths and limitations

This is the first qualitative study to explore the causes of prescribing errors made by different grades of medical as well as non-medical prescribers in an hospital CPOE system setting. Previous research has focussed on prescribing by junior doctors and trainees [5, 29].

The interview guide was piloted in clinical pharmacists only. While they have professional insight into prescribing errors, wider piloting with other professions could have given additional insight. Interviewees were not asked for feedback on the interpretation of their interviews. Our study was carried out in one hospital site operating a single CPOE system, limiting the generalisability of the findings, however our results reflect themes found in the wider literature.

## Conclusion

Medical and non-medical prescribers have similar experience of prescribing errors when using CPOE, with the broad areas of concern aligned with existing published literature about medical prescribing. Causes of electronic prescribing errors are multifactorial in nature and prescribers describe how factors interact to create the conditions errors. Solutions focused on a single factor, such as system design or training, may only result in only limited impact on prescribing errors. While interventions should focus on direct CPOE issues, such as training and design, socio-technical and environmental aspects of practice remain important.

## Electronic supplementary material

Below is the link to the electronic supplementary material.

Supplementary file1 (DOCX 19kb)
