# Supplementary material for: A qualitative study of prescribing errors among multi-professional prescribers within an e-prescribing system
Source: Int J Clin Pharm. 2020 Nov 9;43(4):884–92. doi: 10.1007/s11096-020-01192-0 (PMC8352824; doi:10.1007/s11096-020-01192-0)
Supplement: Supplementary file 1 — Supplementary file1 (DOCX 19kb) [file 11096_2020_1192_MOESM1_ESM.docx]

# Supplement 1

# Semi-structured Interview Questions

The purpose of this interview is to explore your experiences of using electronic prescribing system, your opinions on the factors that contribute to e-prescribing errors, and your opinion and feedback on using electronic prescribing system and system design on the incidence of errors. This will identify the system factors related to errors and consequently prevention strategy can be implemented within the system.

Confidentiality is protected at all times and information analysed or reported from this interview will not allow anyone to recognise you. Patient or colleague information is not required.

The interview will last approximately half an hour and the areas to be covered include a few questions about yourself and your background, followed by the causes and contribution factors related to prescribing errors. Finally, I will ask about system design and feedback that you may think will reduce the errors. The interview will be audio recorded unless you decide not to be recorded. The tapes will be kept securely for five years after the study is completed and then destroyed.

Do you have any questions before starting the interview?

**Part One** Background

Can you tell me a little about yourself?
Job title
Speciality
How long have you been working (experience)?

**Part Two** Discussion about the causes and contribution factors associated with prescribing errors based on case scenarios will be given to the interviewees

What do you think about it? (to explore interviewee’s view)

Why do you think this occurred?

What do you think the contribution factors may have led to this error? (try to encourage the interviewee to explore his/her view on contribution factors) “if some factors not being mentioned move to the next question”

To what extent do you think these factors will contribute the occurrence of an error?

- Patients factor ( complexity of the case)
- Knowing the patient (familiarity of the patient)
- Clinical/therapeutic knowledge
- Work environment including staffing issues, workload and physical environment
- Task factors (lack of protocols, poor ordering layout design or non-routine work)
- Team factors (lack of supervision, poor communication between health care providers and responsibility )
- Computerised system (technology design issues such as use of auto-populate features, dropdown menus picking errors, or inadvertently entering incorrect information)
- Information required when a medication being prescribed
- Organisation and management factors (organizational culture, management styles, coordination, collaboration or training)
- Individual factor (level of experience or mental status)
- Miscellaneous (time of the day)

In general, what do you think possible causes of electronic prescribing errors in inpatient settings?

**Part three:** * Prescribers’ perceptions to prescribing using computerised systems. *Prescribers’ views and concerns to prescribing errors associated such systems. *Feedback on system design to reduce electronic prescribing errors.

To what extent do you think that electronic prescribing system may improve prescribing practice and consequently improve medication safety?

Do you think that using computerised system reduce the incidence of prescribing errors?

Do you have any concerns regarding PICS system?

Has PICS had an impact on you personally/professionally?

Do you have suggestions for PICS design might help to reduce prescribing errors? Try to encourage the interviewee to cover these points:

- User interface
- Prioritising warning messages (alert fatigue)
- Guidelines and protocols to be shown at the point of prescribing (decision support)
- Reliance on decision support (trust)
- Risk factors for individual patient to be displayed
- Computer skills literacy
- Medication history
- Dose adjustment based on laboratory results
- Any other features in PICS may help to reduce prescribing errors

Is there anything you would like to add?

I would like to thank you for your time. This interview has been extremely valuable to the research. If desired a copy of the interview transcript can be sent to you. When the study is completed a summary of the findings will be sent to you if you wish.

## Case studies for discussion:

**Case 1.** Mr. N, a 65-year-old man with history of peptic ulcer disease who is currently taking omeprazole 20 mg (proton pump inhibitor), was admitted to the intensive care unit (ICU) with severe shortness of breath, tiredness and feeling faint. He was diagnosed with acute pneumonia, although pulmonary embolism was considered in the differential diagnosis .The intensivist initiated treatment with both antibiotics and intravenous heparin. Over the next 24 hours, the patient’s clinical status improved, and he was transferred to a medical ward. Before the patient was transferred, a computed tomography chest scan revealed the absence of a pulmonary embolism. After 4 days, hematemesis, hypotension and respiratory distress developed. The patient was intubated for respiratory failure, readmitted to the ICU and given 6 units of blood. Endoscopy showed an actively bleeding peptic ulcer. Intravenous heparin therapy was stopped. Protamine was prescribed as lab result of the partial thromboplastin time (APTT) showed > 150 seconds, and a proton pump inhibitor was prescribed.

**Case 2.** Mrs. N, a 65 year-old female patient with history of G6PD deficiency, was admitted to the hospital with rheumatoid arthritis. On discharge, a house officer has been asked to enter the medications including NSAIDs, folic acid and methotrexate using PICS system. The foundation year 2 was distracted and rushed while he was entering the medication. The warning messages were overridden. As a result, 7.5 mg of methotrexate prescribed daily as well as folic acid.

**Case 3**. Mr. A, 75 years old with chronic renal failure, admitted with myocardial infarction. Enoxaparin 1mg/1kg BD was initiated for acute coronary syndrome. The dose was not adjusted for renal failure as the GFR was <30ml/minute/1.73m2. On day 4 of admission, lab results showed that the haemoglobin level was < 10 g/dl. The clinical pharmacist found that was the result of the inappropriately high dose Enoxaparin.
